# Changing Etiology in Liver Cirrhosis in Sapporo, Japan

**DOI:** 10.5005/jp-journals-10018-1266

**Published:** 2018-05-01

**Authors:** Jong-Hon Kang, Takeshi Matsui

**Affiliations:** 1 Center for Gastroenterology, Teine Keijinkai Hospital, Sapporo, Japan

**Keywords:** Etiology, Japan, Liver cirrhosis.

## Abstract

In Japan, preventive measures and antiviral therapy against acute or chronic viral infection had achieved remarkable progress in the 1980s or later. On the contrary, metabolic syndrome complicated with fatty liver has emerged as a public health concern to date.

In the current study, we attempted to clarify etiological changes in liver cirrhosis treated in a single tertiary institute in Sapporo, Japan, from 1998 to 2016.

Medical records of 1,166 patients (787 males, with mean of 64.9 ± 11.7 years), diagnosed as having liver cirrhosis for 19 years, were retrospectively reviewed to analyze etiology and clinical features.

During the past 10 years, annual numbers of cirrhotic patients with chronic infection of hepatitis B virus (HBV) or hepatitis C virus (HCV) decreased from 50 or more to 20 or less, and alcoholic liver disease or cryptogenic liver injury emerged as major cause of liver cirrhosis. Among 100 cirrhotic patients of unknown cause, nonalcoholic fatty liver disease (NAFLD) occupied almost 50% in 19 observational years.

In order to control the rising trend in NAFLD related with metabolic syndrome, preventive measures including education in society would be required in Japan.

**How to cite this article:** Kang J-H, Matsui T. Changing Etiology in Liver Cirrhosis in Sapporo, Japan. Euroasian J Hepato-Gastroenterol 2018;8(1):77-80.

## BACKGROUND

Liver cirrhosis is regarded as the end stage for all chronic liver diseases regardless of their etiologies. In Japan, the numbers of patients with chronic viral hepatitis have obviously decreased in the new century because of preventive measures performed in the last two decades of the 20th century, such as vaccination and passive immune treatment in fetus against vertical transmission of HBV since 1988, eradication of contamination with viruses in blood products, establishment of single use, or adequate disinfection of medical instruments against horizontal transmission of HCV after the 1990s. In addition, from the beginning of the new century, antiviral therapy has been distributed for controlling sustained infection with hepatotropic viruses; nucleoside or nucleotide analogues for chronic HBV infection or direct acting antivirals (DAA) for chronic HCV infection.

On the contrary, shifting lifestyles on eating and exercise habits have prompted increment of metabolic disorders including fatty liver diseases related with or without excessive alcohol consumption. Of late, there might be rising alcohol consumption in female population and increasing tendency for obesity among middle aged males.^1^

In the current study, we attempted to clarify whether etiological changes in liver cirrhosis could exist or not by fixed-point observation in a single tertiary institute in Sapporo, Japan, during the last 19 years, from 1998 to 2016. And, moreover, we tried to look for upcoming etiological shift in liver cirrhosis in the society concerned.

## MATERIALS AND METHODS

All patients who were hospitalized and diagnosed with liver cirrhosis in a tertiary hospital in Sapporo, Japan, from 1998 to 2016 were retrospectively enrolled in this study. Diagnosis of liver cirrhosis depended on combined clinical features: Symptoms including ascites with or without peripheral edema, results of blood test in serology and chemistry including coagulation system, liver imaging using ultrasonography and/or computed tomography, and histological findings in liver tissue sampled on surgery for hepatocellular carcinoma (HCC).

**Table 1:** **Table 1:** Criteria of etiological diagnosis in liver cirrhosis

*Etiology*		*Criteria*	
HBV		HBsAg/high-level HB core Ab + HBV DNA	
HCV		Anti-HCV	
HCV + HBV		Both of the above	
Alcohol		90 gm ethanol/day over 5 years	
Primary biliary cholangitis		Guideline of the intractable hepatobiliary diseases study group in Japan	
Primary sclerosing cholangitis		Guideline of the intractable hepatobiliary diseases study group in Japan	
AIH		Suspicious or definite in the revised criteria by International AIH Study Group (1998)	
Unknown		Cases noted above were neglected	
NAFLD		US fatty liver among patients with unknown cases	

HBsAg: Hepatitis B virus surface antigen; Ab: Antibody; DNA: Deoxyribonucleic acid

Etiological diagnosis was made in accordance with criteria noted in Table 1.

For the diagnosis of autoimmune hepatitis (AIH) with cirrhotic stage, revised score system by the international AIH study group^2^ was adapted, and the patients in whom there was suspicious or definite scoring were enrolled in the current study.

Primary biliary cirrhosis was also diagnosed using criteria reported from the intractable hepatobiliary diseases study group in Japan.^3^

Diagnosis of HCC was mainly made with liver imaging by ultrasonography, computed tomography, and magnetic resonance imaging for space-occupying lesions in liver, and tumor markers, such as alfa-fetoprotein or protein induced by vitamin K absence/ antagonist-II, were utilized as subsidiary.

In order to evaluate hepatic reserve in patients with liver cirrhosis, medical records in all the subjects were searched and Child-Pugh-Turcotte (CPT) score^4^ was given.

## RESULTS

**Graphs 1A and B:**
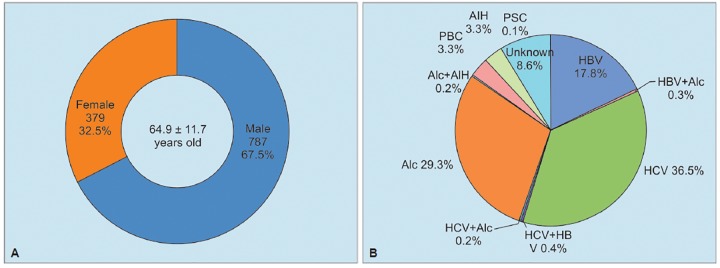
Background (A) and etiology (B) in 1,166 patients with liver cirrhosis diagnosed from 1998 to 2016. Alc: Alcohol; PBC: Primary biliary cholangitis; PSC: Primary sclerosing cholangitis

**Graphs 2A and B:**
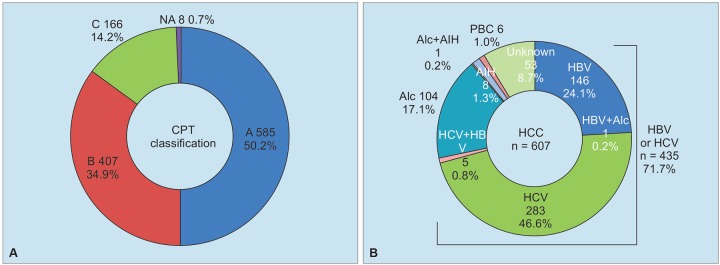
CPT classification (A) and complication of HCC (B) in patients with liver cirrhosis. Alc: Alcohol; PBC: Primary biliary cholangitis; PSC: Primary sclerosing cholangitis

During 19 years, from 1998 to 2016, a total of 1,166 patients with chronic liver disease were diagnosed as having liver cirrhosis. On diagnosis of liver cirrhosis, they were 64.9 ± 11.7 years old, and range was 12 to 95 years. Among them, male patients were 787 (67.5%), and females were 379 (32.5%) (Graph 1A). In accordance with CPT classification, 585 (50.2%) patients were categorized as class A, 407 (34.9%) patients were class B, and 166 (14.2%) patients were class C, among patients with liver cirrhosis (Graph 2A). During follow-up periods or on diagnosis of liver cirrhosis, HCC was diagnosed in 607 (52.1%) patients out of 1,066 cirrhotic patients. Complication of HCC was associated with infection of HCV in 47.4%, HBV in 25.1%, and related with alcoholic liver disease in 17.3%. Out of 607 patients with HCC, 53 (8.7%) cases had no relationship with infection of hepatotropic viruses, alcoholic liver cirrhosis, and autoimmune chronic liver diseases (Graph 2B).

**Graph 3:**
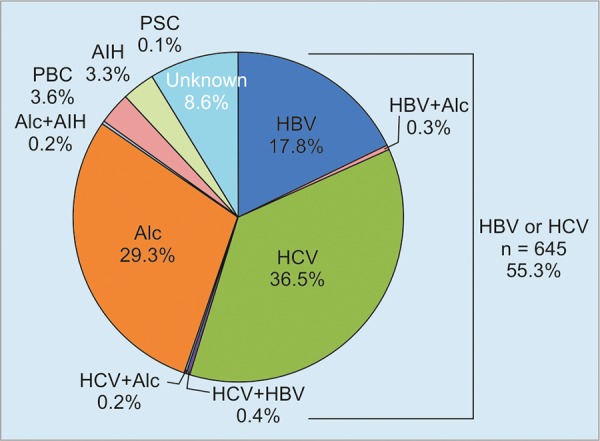
Etiological classification in 1,166 patients with liver cirrhosis. Alc: Alcohol; PBC: Primary biliary cholangitis; PSC: Primary sclerosing cholangitis

As for etiological classification in 1,166 cirrhotic patients, 433 (37.1%) patients were infected with HCV, and 217 (18.6%) were infected with HBV, and 5 had dual infection among 645 (55.3%) patients who had HBV or HCV (Graph 3). Excessive alcohol ingestion alone occupied 29.3% (n = 342) of all cirrhotic patients, ranking 2nd place only after HCV infection, and 100 patients (8.6%) showed unknown etiology. On the contrary, numbers of cirrhotic patients with autoimmune chronic liver diseases, primary biliary cirrhosis, AIH, or primary sclerosing cholangitis were only 78 (6.7%).

**Graphs 4A and B:**
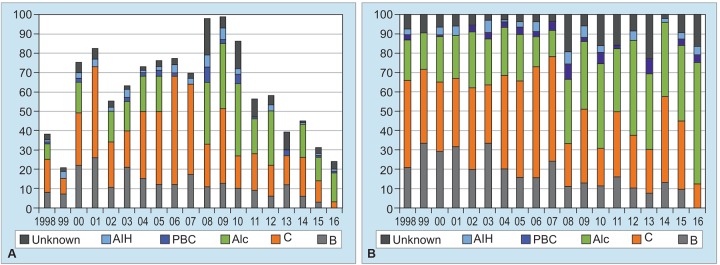
Annual numbers of patients newly diagnosed in every year from 1998 to 2016. (A) Change in annual numbers of patients with liver cirrhosis and in their causes of diseases. (B) Etiological percentage of liver cirrhosis every year. Alc: Alcohol; B: Hepatitis B virus; C: Hepatitis C virus; PBC: Primary biliary cholangitis

Graph 4 shows changing annual numbers of patients who were first given diagnosis of liver cirrhosis from 1998 to 2016. From 1998 until 2009, annual numbers of cirrhotic patients indicated apparent rising tendency, and then, rapid decrease in them was observed in the recent 7 years, from 2010 to 2016 (Graph 4A). Real numbers of patients with HBV showed constant decrease year by year from 2000 to 2016 (Graph 4A), and moreover, share of HBV infection also revealed sustaining drop from 20 to 30% around 2000 to 10% or less after 2010 (Graph 4B).

On the contrary, annual numbers of cirrhotic patients with HCV continued to be 20 or more during 10 years, 2000 to 2009, and then, diminished continuously after 2010. In very strong contrast, alcoholic liver diseases emerged to be the major cause in liver cirrhosis in 2008 or later, in spite of declining total numbers of patients with liver cirrhosis in Graphs 4A and B. Unknown cause in liver cirrhosis had indicated slight increment in patient numbers as well as in its share after 2008 in Graph 4.

**Graphs 5A and B:**
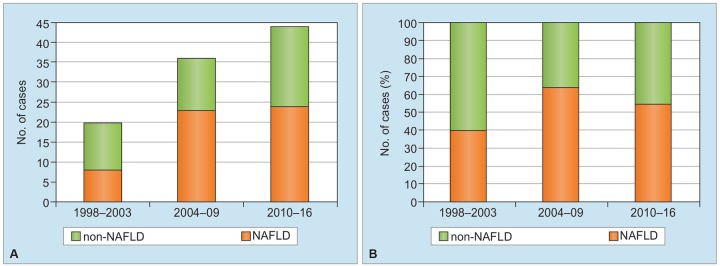
Changes in numbers (A) and percentage (B) of patients with NAFLD among 100 patients with unknown etiology in three periods during the last 19 years

In the current study, 100 patients who had unknown cause of liver cirrhosis were enrolled, and numbers of such patients had gradually risen in these 19 years (Graph 5). Among these patients, numbers of NAFLD significantly increased, when comparing numbers of those in three periods, i.e., 1998 to 2003, 2004 to 2009, and 2010 to 2016, and such patients occupied around 50% in all cases of unknown etiology in every period (Graph 5B).

## DISCUSSION

As for etiology of liver cirrhosis, chronic infection of hepatotropic viruses, such as HBV and HCV demonstrated striking decrease during 19 years observation by a single tertiary hospital in a local city in Japan.

Persistent infection of HBV or HCV was presumed to be controlled by sustained preventive measures against vertical or horizontal transmission in Japan from the 1980s. Moreover, anti-HCV treatment including interferon-based or interferon-free therapy with DAA had been widely accepted from the 2000s in Japan. In addition, treatments with nucleot(s)ide analogs for chronic HBV infection rapidly spread and consequently inhibited disease progression in patients with chronic hepatitis B in the 2000s. According to our current study, excessive alcohol intake and unknown cause had emerged to be major problems related with liver cirrhosis. Among patients with cryptogenic liver cirrhosis, increasing trend in patients with NAFLD has also been demonstrated in this study. In order to control prevalence in NAFLD or nonalcoholic steatohepatitis (NASH), alternative measures deferent from those against infection with hepatotropic viruses would be required.

There are some limitations in the current study. Every case of liver cirrhosis was retrospectively enrolled; therefore, there could be underestimation in numbers of patients with compensative liver cirrhosis. Clinical recognition for NAFLD and NASH was not enough to make appropriate diagnosis around 2000, and patients of “burn-out” NASH might also be difficult to be enrolled in the current study.

In order to prevent disease progression in NASH, health check systems in local societies or workplaces would be remodeled to capture the disease in early phase, and novel diagnostic modality for NASH would be required.

In Asian countries where rapid socioeconomic changes have occurred, fatty liver diseases related with or without alcohol consumption and/or obesity might indicate increasing trend in the very near future, when considering the etiological shift in our institute.

In conclusion, etiological analysis for liver cirrhosis demonstrated that alcoholic liver disease and cryptogenic liver disease including NAFLD or NASH were significantly associated with development of liver cirrhosis in Sapporo, Japan. Because alcohol ingestion or eating habits would be responsible for increment in these chronic liver diseases, educational activities for prevention of these disease setting would be needed in school and community.
